# Detangling ecosystem services: Open‐field manipulation of soil‐dwelling microarthropods provides new opportunities to investigate their effects on nitrogen cycling

**DOI:** 10.1002/ece3.9134

**Published:** 2022-07-19

**Authors:** Veronika Gergócs, Norbert Flórián, Zsolt Tóth, László Sipőcz, Miklós Dombos

**Affiliations:** ^1^ Institute for Soil Sciences Centre for Agricultural Research Budapest Hungary

**Keywords:** ammonium leaching, defaunation, field mesocosms, nitrate leaching, nitrogen cycling, soil microarthropods

## Abstract

1. Soil microarthropods have a pivotal role in soil nitrogen cycling in that they affect microbial decomposers. A high abundance of microarthropods may increase the mobility of inorganic nitrogen ions in the soil, mainly in nitrogen‐limited habitats. However, it is difficult to study ecological processes with small‐sized, soil‐dwelling arthropods. The effects of soil microarthropods on nitrogen cycling have mainly been studied in laboratory microcosm experiments. Therefore, we face many practical issues in investigating these effects under field conditions that remain to be resolved.
2. We developed an open‐field mesocosm setup with growing plants. In a two‐part experiment, spring wheat and grass species were grown in chernozem and sandy soils. Leached ammonium and nitrate ions were measured with percolation lysimeters. Half of the mesocosms included natural assemblages, and the other half included less abundant Acari and Collembola assemblages. The application of nitrogen fertilization assured differences in nitrogen sources.
3. We found a large difference in ammonium and nitrate leaching between the two soil types. In chernozem soil, the leached ion concentrations were higher in mesocosms with more abundant mite and springtail assemblages. The expected patterns were less pronounced in sandy soil. Adding nitrogen fertilizer did not modify the effects of soil microarthropods.
4. Open‐field mesocosms are promising for studying the role of soil‐dwelling mesofauna in ecological processes. We solved the problem of keeping mesofauna abundance lower in treated plots than that in control plots. Plants successfully grew in our semi‐closed systems with functioning percolation lysimeters. The use of the equipment in the experiments in this study helped reveal that the role of soil‐dwelling microarthropods in nitrogen cycling depends on the soil type and not on the application of nitrogen fertilizer.

## Graphical abstract

An open‐field mesocosm was developed to study the role of soil‐dwelling microarthropods in nitrogen cycling. The mesocosms included spring wheat or mixed grass, and a lysimeter collected percolated soil water underneath the systems. We found differences in the leached ammonium and nitrate ion concentrations among mesocosms including higher or lower abundances of mites and springtails.

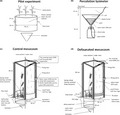

## 1 INTRODUCTION

Nitrogen cycling guarantees that photosynthetic plants obtain nitrogen sources from the soil after microbiota synthesize ammonia by decomposing dead organic materials or fixing atmospheric N_2_ (Bothe et al., 2007). Therefore, the microbiota has a driving role in nitrogen cycling. However, soil bacterial and fungal communities are highly affected by their consumers: especially the mesofauna and microfauna (Ingham et al., 1985; Seastedt, 1984). Mesofauna, mainly microarthropods have a significant indirect role in nitrogen cycling, but their effect on nitrogen‐related soil processes is still not well understood and has been inadequately studied (Filser, 2002; Lakshmi et al., 2020; Soong & Nielsen, 2016).

In most natural habitats, soil‐dwelling mesofauna are a very abundant and species‐rich group dominated by mites (Acari) and springtails (Collembola). High numbers of these invertebrates indicate their significant role in soil ecological processes. The ecological function of mites and springtails could be apprehended through their food utilization. These animals feed partly on dead organic materials including litter and, therefore, contribute directly to decomposition (Heneghan et al., 1999; Seastedt, 1984). However, soil‐dwelling microarthropods may have greater effects on the decomposition through grazing on microbes and transporting their propagules (Verhoef & Brussaard, 1990). Acari and Collembola participate in soil nutrient mobilization by releasing nutrients from microbial cells (Anderson et al., 1983) and excreting nutrient‐rich waste (Griffiths & Bardgett, 1997). Microarthropods also regulate the supply of labile dissolved organic matter, which is a significant limiting factor for soil microbes in nitrogen mineralization (Osler & Sommerkorn, 2007). In addition, mites and springtails help plants take up available nutrients (Ingham et al., 1985; Partsch et al., 2006). The type of habitat also affects the role of soil‐dwelling microarthropods in nitrogen cycling. In nitrogen‐limited habitats (e.g., boreal forests) with higher soil C/N ratios, soil mites and springtails may have greater importance in nitrogen mineralization than in nitrogen‐rich habitats, such as agricultural fields or rainforests (Filser, 2002; Osler & Sommerkorn, 2007).

Microarthropods have a small size (~200–1200 μm) and live a hidden life in the soil. Therefore, their role in nutrient cycling can be studied mainly by manipulating the abundance and composition of their assemblages. These manipulations are primarily realized in microcosm/mesocosm experiments in laboratories or greenhouses (Cole et al., 2004; Partsch et al., 2006; Schon et al., 2011; Wickings & Grandy, 2011). These experiments mostly observed that the presence of soil microarthropods increased nitrogen leaching or mobilization (Bardgett & Chan, 1999; Cole et al., 2004; Cragg & Bardgett, 2001; Peña‐Peña & Irmler, 2018). Although field mesocosm experiments also exist and some of them investigate soil mesofauna, their focal ecological process is mainly decomposition, not nitrogen cycling (Bruckner et al., 1995; Cortet et al., 2003; Zechmeister‐Boltenstern et al., 1998). Field experiments with larger spatial scales investigate more realistic processes than laboratory or greenhouse microcosms/mesocosms (Kampichler et al., 2001).

In addition, most of the microcosm studies on nutrient cycling focus on forest or grassland ecosystems but investigating agricultural fields would also be essential. Nitrogen supply in crop fields is a crucial aspect of farming treatments. However, excessive use of mineral fertilizers may cause soil nitrogen surplus, leading to unwanted nitrogen leaching into deeper soil layers or even into drinking water sources (Sieling & Kage, 2006; Sun et al., 2012). Combining the advantages of laboratory and field mesocosms, Bender and van der Heijden (2015) investigated the role of soil microbiota and microfauna in nutrient leachates in plant–soil system “lysimeters” by manipulating the biomass of soil biota. They measured the concentration of leached ions and compared between agricultural systems with and without animals. They found that soil microfauna reduced nitrogen leaching and improved plant nutrient uptake. This finding contradicts with the results about soil‐dwelling microarthropods causing increased nitrogen leaching in laboratory microcosms (Cole et al., 2004).

The present study aimed to resolve this discrepancy by investigating the role of soil‐dwelling microarthropods in nitrogen cycling in agricultural field conditions with mesocosm systems, including natural and decreased number of soil mites and springtails. According to the definitions by Kampichler et al. (2001), our experimental systems were mesocosms that were partially enclosed soil cylinders under field conditions. In addition, treatments were subtractive since mesocosms comprised manipulated (decreased) soil fauna and perturbative since mineral nitrogen fertilizer affected the systems. However, our mesocosms differed from the definitions by Kampichler et al. (2001), as they did not preserve the full small‐scale spatial complexity of the soil. In the present study, mesocosms also included living plants and a soil water sampler installed below the mesocosms. We compared leached mineral nitrogen compounds among mesocosms including native or decreased soil fauna.

In the field, it is challenging to ensure similar conditions in treated and control mesocosms, including manipulated soil, living plants, and buried soil water samplers. The present study comprised a pilot experiment and two other experiments. In the pilot experiment, we tested the manipulation of soil faunal abundance and the function of a soil water sampler (lysimeter). Based on the pilot test, we used a developed mesocosm system in the main experiments. We expected that the defaunated mesocosm systems would contain less abundant Acari and Collembola assemblages than the control mesocosms. First, according to the results of previous microcosm experiments (e.g., Cragg & Bardgett, 2001), we hypothesized that lower nitrate and ammonium concentrations would leach out from the mesocosms with lower microarthropod abundance. Second, we hypothesized that nitrogen fertilization and soil type would modify the effects of microarthropod abundance on leaching. We also expected that the nitrogen uptake of plants and the biomass of soil microbes would be higher at more abundant soil Acari and Collembola assemblages.

## 2 MATERIALS AND METHODS

### 2.1 Study site

The study was conducted in two different locations, at the periphery of Nagyhörcsök village (Hungary, 46°51′56.62″N, 18°31′08.41″E) and Őrbottyán town (Hungary, 47°40′10.15″N, 19°15′12.15″E). These locations belong to the experimental fields of the Centre for Agricultural Research, Hungary. The soil types were calcareous sandy soil [WRB classification: Mollic Umbrisol (Arenic)] in Őrbottyán and calcareous chernozem (WRB: Calcaric Phaeozemet) in Nagyhörcsök.

### 2.2 Pilot experiment (2019, maize)

#### 2.2.1 Experimental design

**FIGURE 1:**
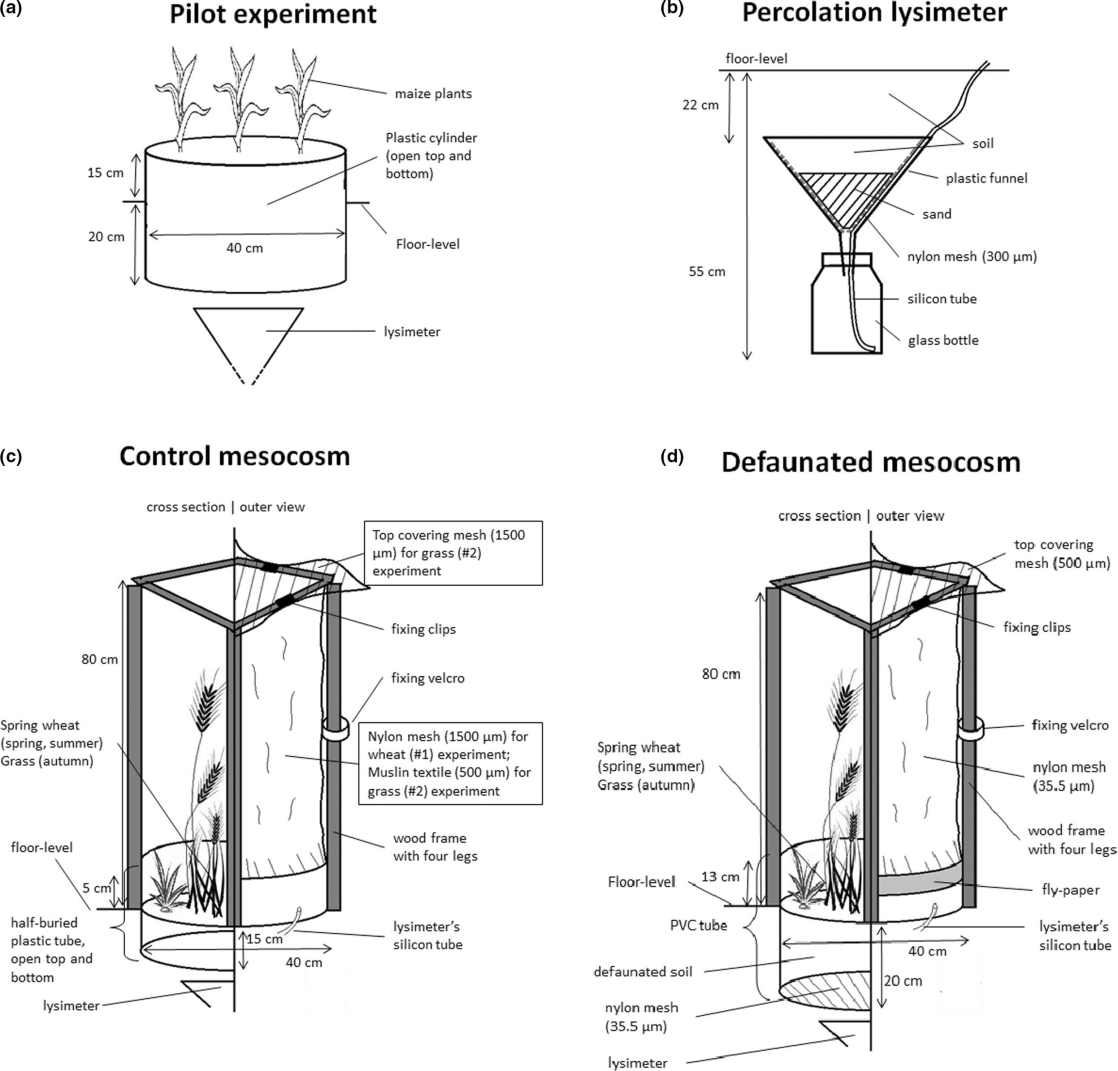
Structure of mesocosms and percolation lysimeters in the pilot and #1–2 experiments.

Two weeks before the pilot experiment, four cylinder holes (15 cm depth and 40 cm diameter) were dug 1.5 m apart in a line, and the removed soil was placed into plastic bags (size: 30 × 40 cm). Soil bags were immediately taken into a deep freezer and kept at −20°C for 2 weeks to defaunate the soil. As the soil was collected in May, soil‐dwelling arthropods were considered active and not prepared for an extreme freeze. Plastic cylinders (with an open bottom) were placed into the holes (35 cm height and 40 cm diameter, buried at 20 cm depth) to prevent the soil mesofauna from entering the spot (Figure 1a).

After 2 weeks, on May 16, 2019, a percolation lysimeter (Figure 1b; Derome et al., 1991) was established in the bottom of each hole. Briefly, a percolation lysimeter is composed of a glass jar (750 ml, 12 cm height and 9 cm diameter) and a plastic funnel (height and diameter: 22 cm) placed above that. A silicon tube comes from the funnel into the jar. In the funnel, 300 cm^3^ of sand (particle size 0.5–1.2 mm) was placed on a nylon mesh (size: 300 μm). Nylon mesh is placed above the silicon tube, preventing the sand from entering the jar. All components were buried to 55 cm depth; therefore, approximately 20 cm of soil was above the funnel. The silicon tube hung out from the soil. Percolated water was sampled by a syringe from the glass jar through the silicon tube. After lysimeters were buried, the thawed soil was placed back into the four plastic cylinders (Figure 1a). Then, eight maize (*Zea mays*, L.) seeds were seeded along the diameter of the mesocosms.

We tested whether defaunation and soil removal/replacement procedures affect the leaching of soil ammonium and nitrate ions. Therefore, four additional lysimeters and plastic cylinders were established next to the defaunated systems without freezing the soil within the cylinder. The soil was removed and placed back into the cylinder and after maize was seeded.

#### 2.2.2 Sampling

Three weeks later (on June 4), there was substantial rainfall, and the percolation lysimeters were sampled. Three months later, soil fauna were sampled using a metal corer (cylinder, 8 cm diameter and 8 cm depth). There was no considerable additional rainfall in this season needed to sample the lysimeters.

#### 2.2.3 Results of the pilot experiment

Maize plants grew similarly in the treated and control plots (data not shown). After 1 month, lysimeters showed similar nitrate and ammonium ion concentrations in the defaunated and control plots (Table S1). However, some lysimeters did not include water after the rainfall in chernozem soil. After 3 months, the soil fauna were very similar between the control and the defaunated plots (Figure S1). In the defaunated plot, Mesostigmata were significantly more abundant in sandy soil and Endeostigmata were more abundant in chernozem soil. According to the experiences of the pilot experiment, percolation lysimeters worked properly. However, manipulation of mesofauna abundance had to be substantially improved.

### 2.3 Experiment #1 (2020, spring wheat)

#### 2.3.1 Experimental design

On February 25, 2020, four blocks were established in each location, two of which were nitrogen‐fertilized (Figure S2). Phosphate and potassium fertilizers were added to all blocks according to the demand of spring wheat (*Triticum aestivum*, L.) on a given location: chernozem soil (Nagyhörcsök); 63 kg/ha superphosphate and 63 kg/ha potassium carbonate; sandy soil (Őrbottyán): 89 kg/ha superphosphate and 59 kg/ha potassium carbonate. Nitrogen fertilizer was added later [chernozem soil: 155 kg/ha calcium ammonium nitrate (CAN) and sandy soil: 113 kg/ha CAN]. On the same day, for defaunation, approximately 402 L of soil was collected into plastic bags from the 10–20 cm topsoil layer within the blocks. Plastic bags were brought into a deep freezer (−20°C) for 2 weeks. In February, the air temperature was unusually high (average daily temperature was consistently above 5°C), and soil‐dwelling animals were considered to be already active.

After 2 weeks, spring wheat was seeded in all blocks (23 g/m^2^). On the same day, 32 mesocosms were established in two parallel rows within the blocks (Figure S1). There were two types of mesocosms: control and defaunated mesocosms (Figures 1c,d). In each block, four‐four plots were set up in two rows for the mesocosms. The two rows were 2 m apart, and within a row, the plots were 0.6 m and 1.1 m apart from each other, alternating, which was enough for the mesocosms not to shadow each other. In addition, a 2‐m‐wide buffer zone was established around the blocks (Figure S2).

Before the building of mesocosms, a percolation lysimeter was buried underneath each plot (Figure 1b). Then, for establishing defaunated mesocosms (Figure 1d), a PVC tube (33 cm height and 40 cm diameter) was placed to 20 cm depth above the lysimeters. The bottom of the tube was covered by a nylon mesh (35.5 μm) keeping away soil‐dwelling microarthropods (Dittmer & Schrader, 2000). Thawed (defaunated) soil was placed into each tube at 20 cm height, until the soil surface. In addition, 80‐cm‐high, four‐legged wood frame was built for each mesocosm. In addition to the bottom of the PVC tube, a nylon mesh cover kept soil‐dwelling microarthropods away from the top of the tube in defaunated mesocosms (Figure 1d). This nylon mesh cylinder (size: 35.5 μm) was fixed to the top edge of the PVC tube and to the wood frame. The top of the wood frame was covered with another nylon mesh (size: 500 μm).

For control mesocosms (Figure 1c), the previously dug soil was placed back above the lysimeters. The plot was surrounded by a 20‐cm‐high plastic tube (15 cm deep in the soil) making a 40‐cm‐diameter cylinder. A same wood frame was built for each control mesocosm such as to defaunated mesocosms. Similar light conditions must have been assured for plants in both mesocosm types; therefore, the control mesocosms were also covered with a nylon mesh cylinder (size: 1500 μm), which was permeable for microarthropods. However, no covering nylon mesh was placed on the top of the wood structure in control mesocosms (Figure 1c). The conformation of the mesocosms allowed us to collect samples from inside.

The last steps were to seed spring wheat and to put nitrogen fertilizer into the mesocosms. Finally, a 5‐cm‐wide fly paper stripe was fixed on the outer wall of the PVC tube to prevent arthropods from crawling up to the mesocosms.

#### 2.3.2 Sampling and analyses

Soil samples were collected in 2 months to reveal the changes in soil faunal abundance and soil water ion concentration over time (Table 1). On June 18 and 19, 2020, soil fauna were sampled using a hand soil corer (~20 cm^3^). Six subsamples were collected from eight mesocosms in each soil type (two samples from all treatment combinations: defaunated and fertilized, control and fertilized, defaunated and unfertilized, control and unfertilized). Three subsamples from 0–8 cm and three subsamples from 8–16 cm depth were taken. Then, these six subsamples were pooled before extraction. In June, lysimeters remained empty after heavy rainfall (76–81 mm) due to the previous dry period. Therefore, 3 L of tap water were added to all plots in chernozem soil, and 6 L were added to sandy soil. Tap water was presumed not to include ammonium and nitrate ions. If it included some minimal N content, it would equally affect all the mesocosms. After 1 h, percolated water was collected (100 ml) with a syringe from each lysimeter and stored at −20°C until chemical analyses.

**TABLE 1:** Timeline of the experiments (Exp.) in 2020 in both locations.

Exp.	Month	Activity	Sampling
#1	February	Freezing soil	
	March	Building mesocosms, seeding spring wheat, fertilization	
	June		Soil water, fauna
	July		Soil water, fauna, soil chemistry, plant nitrogen content, wheat yield, microbiota
#2	August	Freezing soil	
	September	Modifying and repairing mesocosms, seeding mixed grass, fertilization	
	October		Soil water
	November		Fauna, grass biomass

On July 16 and 17, after spring wheat was harvested in blocks, sampling of soil fauna and percolated water was repeated. Soil fauna samples were collected from eight other mesocosms compared with the June sampling. In addition, 300 g of soil samples was collected from the 5 cm topsoil layer of the mesocosms for soil chemical analyses, and 100 g of soil samples was collected for microrespiration analyses. Furthermore, vegetative parts and ears of the wheat were sampled in each mesocosm. Soil samples of chemical analyses and samples of wheat were air‐dried and stored at room temperature. Microbial samples were stored at 4°C until analyses.

Fauna samples were extracted using a Berlese funnel for 1 week without temperature gradient and kept in 70% methanol until determination. Most microarthropods were springtails and mites. Mite specimens were sorted into different groups [based on (Krantz & Walter, 2009)] and enumerated: order Mesostigmata (mainly cohort Gamasina), cohort Heterostigmatina (“Heterostigmata,” with Pygmephoridae, Tarsonemidae), suborder Endeostigmata (Nanorchestidae), suborder Prostigmata (Eupodidae, Tydeidae, Ereynetidae) and cohort Astigmata (Acaridae and Histiostomatidae). Collembola were not sorted into groups due to their low abundance.

Determination of the exchangeable ammonium and nitrate contents of the soil and the total nitrogen content of the plant tissue was conducted according to steam distillation methods (Bremner, 2016; Bremner & Keeney, 1965). The soil water ammonium and nitrate concentrations were determined by the Hungarian standards MSZ ISO 7150‐1 and MSZ 1484‐13, respectively.

Microbial biomass was estimated by substrate‐induced respiration (Anderson & Domsch, 1978; Holden & Treseder, 2013; Kaiser et al., 1992). Three subsamples (~3 × 15 cm^3^) were obtained from each plot from the 10 cm topsoil layer and were mixed in a plastic bag representing a given mesocosm. A 20 g sample was used to measure the gravimetric water content via oven drying at 105°C two times for 3 h. To determine CO_2_ production, 2 g of soil was freshly sieved (mesh size: 2 mm), then weighed and incubated for 3 days. Then, 200 μl of glucose solution (0.04 g/ml) was added to 2 g of soil. After a 3‐h incubation, the produced CO_2_ was determined by a gas chromatograph (FISONS GC8000) with a flame ionization detector after methane conversion. Gas samples were taken with a 250 μl syringe from the vessels. The peak of the CO_2_ measurement was recorded, and the substrate‐induced respiration was calculated (Ananyeva et al., 2011). We presumed that changes in soil microbial communities appearing after thawing would diminish over time in the field (Aanderud et al., 2013).

### 2.4 Experiment #2 (2020, grass)

#### 2.4.1 Experimental design

In experiment #2, the two slightly modified mesocosm types of experiment #1 were used with grass species instead of wheat. In August, residues of wheat plants were removed from plots, and the soil from the defaunated mesocosms was frozen again. On September 2 and 3, the experimental areas were tilled by a rototiller sidestepping the mesocosms. Fertilizers were spread in chernozem soil (63 kg/ha superphosphate and 63 kg/ha potassium carbonate) and in sandy soil (150 kg/ha superphosphate and 300 kg/ha potassium carbonate). Nitrogen fertilizer was added only to “fertilized blocks” (chernozem soil: 155 kg/ha CAN and sandy soil: 150 kg/ha CAN). Then, a grass seed mixture (40% *Festuca rubra*, L., 20% *Lolium perenne*, L., 20% *Festuca heterophylla*, Lam., and 20% *Festuca arundinacea*, Schreb.) was seeded at 50 g/m^2^.

Defaunated mesocosms had the same structural composition as in experiment #1. Meanwhile, control mesocosms were improved in two ways (Figure 1d). First, the covering mesh cylinder was changed to a muslin textile. This textile was still permeable for microarthropods and allowed an equivalent amount of light into the mesocosms as for defaunated ones. Second, the wood frame was covered by a nylon mesh (mesh size: 1500 μm) at the top (Figure 1d). This top covering nylon mesh was needed to equate the amount of rainfall in the two types of mesocosms since the covering mesh partly prevented the rain from falling into the mesocosms. In addition, top covering mesh may have modified the temperature and humidity inside the mesocosms, and these conditions should have been equalized between the control and defaunated mesocosms.

#### 2.4.2 Sampling

After heavy rainfall (80–85 mm) in October, lysimeters contained enough water, and 100 ml samples were collected on October 26 and stored at −20°C until analyses. Other samplings were conducted on November 11. Fresh grass biomass was harvested (cut down at 3 cm), taken into plastic bags, and later air‐dried and weighed. Soil fauna were sampled using the same soil corer as in experiment #1 from all plots. Methods of fauna extraction and chemical analyses of soil water were the same as in experiment #1.

### 2.5 Statistical analyses

Abundances of taxonomic groups were standardized for 100 cm^3^ soil volume and then log (*x* + 1)‐transformed. For June and July (experiment #1), we had fewer data for microarthropods; therefore, effects of defaunation and fertilization were analyzed with the Mann–Whitney–Wilcoxon test among defaunated and control mesocosms and separately among fertilized and unfertilized mesocosms per soil type in R version 3.6.1. (wilcox.test[] function). For experiment #2, two‐way ANOVA models were performed for abundances among factors fertilized/unfertilized and faunated/defaunated per soil type (package *stats*, lm[] function, R Core Team). Log‐abundances were performed in barplots using the ddply() function [package: *plyr*, (Wickham, 2011)].

Exchangeable soil ammonium and nitrate content, plant biomass, plant nitrogen content, SIR, and soil water ammonium and nitrate concentration were analyzed using three‐way ANOVA models with the factor levels of sandy/chernozem soil type (Soil), fertilized/unfertilized (Fertilization) and control/defaunated (Fauna) as the following formula: response variable ~ Fertilization * Fauna * Soil. In the case of experiment #1, models of soil water ions included the factor month. However, month did not have any significant effects in the models; therefore, we presented those models that do not include the factor month. The analyses were performed in R (package *stats*, lm() function, R Core Team). Eight repetitions belonged to all treatment levels; however, 1–3 data were missing in the case of the soil water samples. Although response variable data were log‐transformed for better model diagnostic results, the tables include untransformed average and standard deviation data. The models were tested for normal distribution, outliers, and homoscedasticity (“plot()” function), and the models with satisfying diagnostics were displayed. To compute post hoc tests within the three‐way ANOVA models, we used the functions grouped_by() and anova_test() (package *statix*).

## 3 RESULTS

### 3.1 Experiment #1 (2020, spring wheat)

**FIGURE 2:**
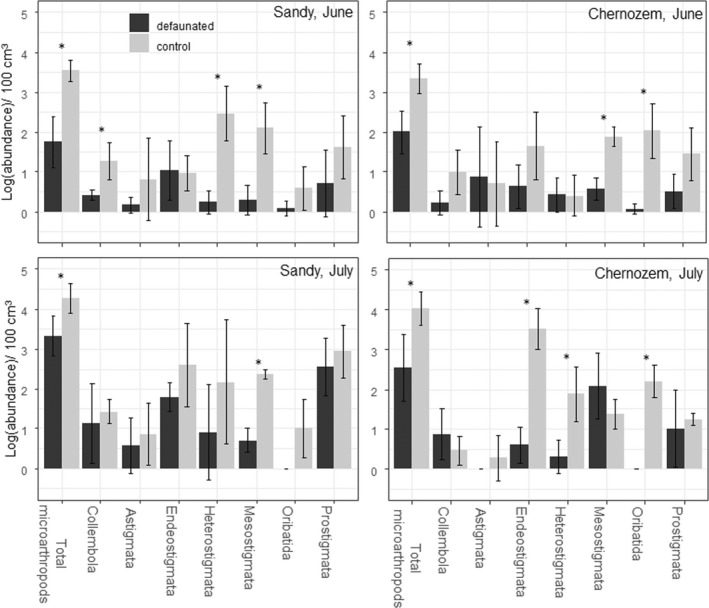
Average (±SD) microarthropod log‐abundances in the two soil types in June and July 2020 in experiment #1. Significant differences between control and defaunated mesocosms in Mann–Whitney–Wilcoxon test (*n* = 4): *p < .05.

The total number of Acari and Collembola specimens was mainly not affected by fertilization (Table S2). Only Collembola (*W* = 0, *p* = .029) and Prostigmata (*W* = 0, *p* = .029) had significantly higher abundances in fertilized mesocosms in July. Microarthropod abundances were significantly lower in defaunated mesocosms than in control systems (Figure 2). However, group abundances exhibited various patterns among soil types and months. In sandy soil, Collembola, Heterostigmata, and Mesostigmata (*W* = 0, *p* = .029) were more abundant in control mesocosms than in defaunated mesocosms in June. Nonetheless, after a month, only Mesostigmata were significantly less abundant in defaunated mesocosms (*W* = 0, *p* = .029). In chernozem soil, Mesostigmata and Oribatida (*W* = 0, *p* = .029) were significantly less abundant in defaunated mesocosms in June. In July, Endeostigmata, Heterostigmata, and Oribatida (*W* = 0, *p* = .029) were less abundant in defaunated mesocosms. The other groups exhibited similar abundances in the two types of mesocosms.

The ammonium and nitrate contents of the soil water exhibited similar patterns between June and July (Table 2). Leached nitrate concentrations were higher in the fertilized mesocosms than in the unfertilized mesocosms in both soil types (*F* = 187.0, *p* = 2.2 × 10^−16^). In fertilized mesocosms of chernozem soil, nitrate leaching tended to be higher in control mesocosms than in defaunated mesocosms. In sandy soil, there was higher nitrate leaching in defaunated than in control mesocosms (fertilized: *F* = 6.7, *p* = .011, unfertilized: *F* = 20.8, *p* = 1.3 × 10^−5^). Leached ammonium concentrations were not significantly affected by fertilization (*F* = 1.0, *p* = .319) (Table 3). There was a significant interaction between soil type and defaunation (*F* = 23.0, *p* = 6.3 × 10^−6^). In sandy soil, defaunated mesocosms exhibited higher ammonium concentrations than control mesocosms (*F* = 25.4, *p* = 1.9 × 10^−6^).

**TABLE 2:** Averages and standard deviations of the variables measured in the two soil types in experiment #1.

Variable	Month	Sandy soil			
		Fertilized		Not fertilized	
		Defaunated	Control	Defaunated	Control
${{\mathrm{NO}}_{3}}^{-}$ (mg/L)	June	336.63 ± 211.56	137.39 ± 52.42	40.42 ± 33.76	4.57 ± 2.30
	July	154.95 ± 66.34	58.47 ± 42.91	37.55 ± 18.37	9.06 ± 3.24
${{\mathrm{NH}}_{4}}^{+}$ (mg/L)	June	0.13 ± 0.07	0.11 ± 0.09	0.09 ± 0.09	0.04 ± 0.03
	July	0.15 ± 0.12	0.07 ± 0.08	0.19 ± 0.15	0.06 ± 0.05
Soil ${{\mathrm{NO}}_{3}}^{-}$ (mg/kg)	July	13.62 ± 4.23	5.59 ± 1.58	6.63 ± 3.66	3.35 ± 1.76
Soil ${{\mathrm{NH}}_{4}}^{+}$ (mg/kg)	July	3.46 ± 0.67	2.42 ± 0.21	2.71 ± 0.49	2.47 ± 0.53
Plant N content (m/m%)	July	0.98 ± 0.26	0.84 ± 0.10	0.86 ± 0.16	0.62 ± 0.08
Wheat yield (g)	July	5.41 ± 2.62	44.13 ± 9.82	6.37 ± 3.95	45.14 ± 11.32
SIR (μg CO_2_‐C/g soil/h)	July	6.09 ± 1.29	4.98 ± 0.88	5.42 ± 1.05	4.82 ± 0.43

Variable	Month	Chernozem soil			
		Fertilized		Not fertilized	
		Defaunated	Control	Defaunated	Control
${{\mathrm{NO}}_{3}}^{-}$ (mg/L)	June	219.30 ± 139.89	268.49 ± 99.88	55.32 ± 50.77	3.92 ± 3.30
	July	171.05 ± 104.76	342.53 ± 36.00	17.94 ± 12.25	16.30 ± 8.39
${{\mathrm{NH}}_{4}}^{+}$ (mg/L)	June	0.08 ± 0.02	0.31 ± 0.20	0.13 ± 0.07	0.23 ± 0.20
	July	0.05 ± 0.02	0.16 ± 0.14	0.05 ± 0.01	0.08 ± 0.02
Soil ${{\mathrm{NO}}_{3}}^{-}$ (mg/kg)	July	12.76 ± 19.63	9.76 ± 4.82	1.53 ± 0.02	2.61 ± 0.56
Soil ${{\mathrm{NH}}_{4}}^{+}$ (mg/kg)	July	4.07 ± 1.41	2.93 ± 0.54	1.98 ± 0.32	2.24 ± 0.26
Plant N content (m/m%)	July	0.78 ± 0.31	0.69 ± 0.08	0.22 ± 0.05	0.53 ± 0.15
Wheat yield (g)	July	7.67 ± 5.17	26.92 ± 7.68	9.35 ± 2.56	31.98 ± 9.72
SIR (μg CO_2_‐C/g soil/h)	July	4.06 ± 0.93	3.52 ± 0.34	2.70 ± 0.76	4.30 ± 0.59

Abbreviation: SIR, substrate‐induced respiration.

**TABLE 3:** Coefficients of the ANOVAs with the variables assessed in experiment #1.

Experiment #1	${{\mathrm{NO}}_{3}}^{-}$ (mg/L)	${{\mathrm{NH}}_{4}}^{+}$ (mg/L)	Soil ${{\mathrm{NH}}_{4}}^{+}$ (mg/kg)	Soil ${{\mathrm{NO}}_{3}}^{-}$ (mg/kg)	Plant N content (m/m%)	Wheat yield (g)	SIR (μg CO_2_‐C/ g soil/h)
	*R* ^2^ = .69	*R* ^2^ = .20	*R* ^2^ = .51	*R* ^2^ = .60	*R* ^2^ = .61	*R* ^2^ = .82	*R* ^2^ = .58
Intercept: unfertilized, defaunated, chernozem	2.91***	−2.63***	0.67***	0.43*	0.22***	9.35***	2.70***
Fertilization (fertilized)	2.02***	−0.19	0.68***	1.43***	0.56***	−1.67	1.36**
Fauna (control)	−1.18***	0.58	0.13	0.51	0.31***	22.64***	1.60***
Soil (sandy)	0.52	0.26	0.31**	1.36***	0.63***	−2.98	2.72**
Fauna (control): Soil (sandy)	−0.45	−1.70**	−0.22	−1.18**	−0.56***	16.12**	−2.20***
Fertilization (fertilized): Soil(sandy)	−0.22	0.31	−0.44**	−0.65	−0.44***	0.71	−0.70
Fauna(control): Fertilization (fertilized)	1.91***	0.74	−0.42**	−0.20	−0.40**	−3.39	−2.14***
Fertilized:Control:Sandy	−1.18	−0.34	0.17	−0.01	0.50**	3.35	1.64

*Note*: Ion concentrations were log‐transformed. Factor levels in brackets are those levels which are compared with the reference level.

Abbreviations: *R* ^2^, coefficient of determination; SIR, substrate‐induced respiration.

*p < .05, **p < .01; ***p < .001.

The exchangeable ammonium and nitrate contents of the soil were higher in fertilized mesocosms and in sandy soil (Table 3). The nitrate ion content of the sandy soil was higher in the defaunated mesocosms than in the control mesocosms (*F* = 18.0, *p* = 8.2 × 10^−5^). Within defaunated mesocosms, sandy soil had higher nitrate concentration than chernozem soil. The nitrogen content of wheat plants was higher in fertilized (*F* = 37.6, *p* = 9.2 × 10^−8^) mesocosms. In the unfertilized chernozem soil, the plant nitrogen content was higher in control mesocosms (*F* = 13.3, *p* = .0006). The yield of spring wheat was much higher in control than in defaunated mesocosms in both soil types (*F* = 262.7, *p* = 2.2 × 10^−16^) (Tables 3 and 4). Substrate‐induced respiration (SIR) of the soil microbiota exhibited higher values in sandy, defaunated mesocosms (*F* = 8.3, *p* = .006). Except for the unfertilized chernozem soil, where the SIR values were higher in control mesocosms (*F* = 13.0, *p* = .0007).

**TABLE 4:** Averages and standard deviations of the variables measured in the two soil types in experiment #2.

Variable	Month	Sandy soil			
		Fertilized		Not fertilized	
		Defaunated	Control	Defaunated	Control
${{\mathrm{NO}}_{3}}^{-}$ (mg/L)	October	283.24 ± 77.40	344.18 ± 37.79	237.45 ± 65.33	259.43 ± 121.19
${{\mathrm{NH}}_{4}}^{+}$ (mg/L)	October	0.05 ± 0.02	0.11 ± 0.09	0.07 ± 0.02	0.18 ± 0.12
Grass biomass (g)	November	10.00 ± 4.68	11.94 ± 3.20	10.56 ± 1.58	9.26 ± 2.22

		Chernozem soil			
		Fertilized		Not fertilized	
		Defaunated	Control	Defaunated	Control
${{\mathrm{NO}}_{3}}^{-}$ (mg/L)	October	217.60 ± 65.05	350.29 ± 48.14	73.56 ± 48.88	117.75 ± 68.26
${{\mathrm{NH}}_{4}}^{+}$ (mg/L)	October	0.03 ± 0.01	0.04 ± 0.01	0.03 ± 0.01	0.08 ± 0.04
Grass biomass (g)	November	10.44 ± 5.45	1.98 ± 2.12	6.43 ± 1.03	1.69 ± 1.12

### 3.2 Experiment #2 (2020, mixed grass)

**FIGURE 3:**
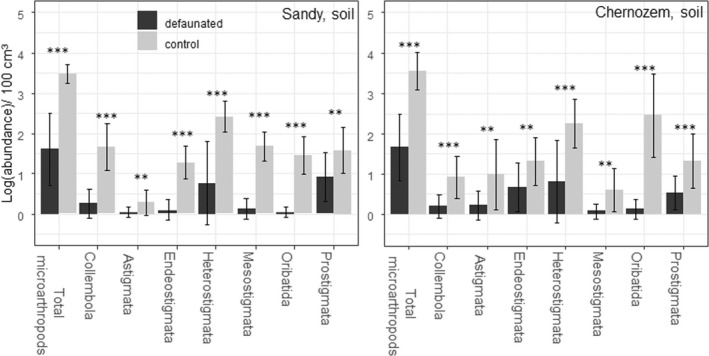
Average (±SD) log‐abundance data of microarthropod groups in the two soil types in experiment #2 (*n* = 16). Significant differences between control and defaunated mesocosms in two‐way ANOVA (factor fertilization was insignificant: Table S2). *p < .05, **p < .01; ***p < .001.

Defaunation was more effective in experiment #2 than in #1. The mean soil‐dwelling microarthropod abundance was significantly lower in defaunated mesocosms than in control mesocosms after 2.5 months (Figure 3). The fertilization showed no effect on abundances (Table S2).

Fertilization slightly affected grass biomass (*F* = 4.3, *p* = .042). In sandy soil, grass biomass was similar in the two mesocosm systems (*F* = 0.1, *p* = .766) (Table 4). However, in chernozem soil, plant biomass was much lower in the control than in defaunated mesocosms (*F* = 36.6, *p* = 1.3 × 10^−7^). Nitrate concentrations of soil water were higher in sandy soil and in fertilized mesocosms than in chernozem soil (*F* = 18.4, *p* = 9.5 × 10^−5^) and unfertilized mesocosms (*F* = 38.6, *p* = 1.5 × 10^−7^), respectively (Table 5). The leached nitrate ion concentration was higher in control mesocosms than in defaunated mesocosms (*F* = 5.3, *p* = .026). There was a significant interaction between soil type and fertilization (*F* = 13.3, *p* = .0007) meaning that the fertilization had higher effect in chernozem soil than in sandy soil. Fertilization slightly affected ammonium ion concentration in November (*F* = 5.1, *p* = .030). The ammonium concentrations of soil water were significantly higher in control mesocosms than in defaunated mesocosms (*F* = 8.9, *p* = .005) (Table 5).

**TABLE 5:** Coefficients of the ANOVAs with the variables assessed in experiment #2.

Experiment #2	${{\mathrm{NO}}_{3}}^{-}$ (mg/L)	${{\mathrm{NH}}_{4}}^{+}$ (mg/L)	Grass biomass (g)
	*R* ^2^ = 0.57	*R* ^2^ = 0.35	*R* ^2^ = 0.58
Intercept: unfertilized, defaunated, chernozem	4.01***	−3.47***	6.43***
Fertilization (fertilized)	1.33**	−0.01	4.01*
Fauna(control)	0.62*	0.72*	−4.74**
Soil (sandy)	1.42***	0.83	4.13
Fauna(control): Soil(sandy)	−0.64	−0.05	3.44
Fertilization (fertilized): Soil(sandy)	−1.15**	−0.35	−4.58*
Fauna(cont.): Fertilization (fertilized)	−0.11	−0.58	−3.72
Fertilized:Control:Sandy	0.36	0.42	6.97*

*Note*: Ion concentrations were log‐transformed. Factor levels in brackets are those levels which are compared with the reference level.

Abbreviation: *R* ^2^ = coefficient of determination.

*p < .05; **p < .01; ***p < .001.

## 4 DISCUSSION

### 4.1 Mesocosm technology

In the present study, we built and tested a mesocosm system in which the abundance of soil‐dwelling microarthropods was manipulated (decreased) to investigate the role of microarthropods in nitrogen cycling. Field conditions provided three challenges. The first challenge was maintaining a significant difference in microarthropod abundance among defaunated and control mesocosms. The second was maintaining similar conditions for developing plants in treated and control mesocosms. The third challenge was extracting adequate samples from percolation lysimeters.

Two steps are needed to keep the abundance of soil‐dwelling animals low in a semi‐closed field system. First, animals should be killed in the soil, while preventing extreme changes in the soil structure. Second, microarthropods should be kept outside the mesocosms. In agricultural fields, the topsoil layer is usually disturbed due to tillage. Therefore, removing and manipulating the topsoil layer seem to be ordinary in such an environment. Almost 1 m^3^ of soil was defaunated in the present experiment. Using chemicals (Eisenhauer et al., 2010) would have negatively affected plants. Sieving and drying (Schon et al., 2011; Sechi et al., 2014) were rejected to avoid serious modification of the soil structure. Therefore, freezing was used to defaunate the soil (Endlweber & Scheu, 2006; Haase et al., 2008; Ke & Scheu, 2008; Kreuzer et al., 2004).

The efficiency of defaunation was more successful in experiment #2 than in experiment #1. Efficiency might be affected by the length of freezing and the date that soil samples are collected in the field. The soil samples of experiment #2 were kept longer (4 weeks) in deep freezers than those in experiment #1 (2 weeks). In addition, soil samples were collected in experiment #1 in February (mild winter time) and in experiment #2 in July (hot and dry summer time). Longer freezing and summer‐time collection may have made the defaunation more effective in experiment #2. Lenoir et al. (2007) reported that freezing soil samples for 3 days resulted in microarthropod eggs remaining alive. Soil microarthropods seemed to be more vulnerable to freezing in the summer than at the end of the winter. However, defaunation could be improved with alternating freezing and thawing periods for several weeks, as in other microcosm experiments (Gestel et al., 2003; Mitschunas et al., 2006; Wolfarth et al., 2011). Drying and freezing the soil would be another effective solution for defaunation (Cortet et al., 2003); however, then, control mesocosms should have also been treated and then refaunated, providing additional time and energy‐consuming tasks. We think combination of long freezing period (2–3 months) with 2–3 thawing occasions would cause the less destruction on soil structure but greatest harm in soil mesofauna.

Freezing or alteration of freezing and thawing may also modify the soil nitrate and ammonium content (Henry, 2007). In experiments investigating this phenomenon, after a freezing period, a large amount of water was added to leach out the nutrients (Joseph & Henry, 2008). We did not irrigate the mesocosms after thawing. In addition, higher soil nitrate content after thawing compared with unfrozen system may happen because of the decreased plant uptake after frost (Campbell et al., 2014) but our system did not include plants just after thawing, seeds had to germinate first. According to our pilot experiment, the differences caused by freezing in soil nitrate content may become negligible after several months.

The second step is to keep microarthropods outside the mesocosms under field conditions. In traditional litter‐bag experiments, microarthropods are usually excluded from litter‐bags with a mesh cover; however, these systems did not include living plants and estimate the speed of organic matter decomposition (Dittmer & Schrader, 2000; Kampichler & Bruckner, 2009). The most significant novelty of the present experiment was to set low abundances of soil‐dwelling microarthropods while growing plants in a field‐scale observation system. Without the 35‐μm mesh net, microarthropod abundances in defaunated soils became similar to the control mesocosms in the pilot experiment. In experiment #2, soil‐dwelling Acari and Collembola were significantly less abundant in defaunated mesocosms. Therefore, the 35‐μm mesh net effectively excluded soil microarthropods from the mesocosms. Consequently, when the first challenge was incomplete, it may have been caused by the inappropriate defaunation method, not by the failure to prevent microarthropods from entering the mesocosms. This technique of keeping out the fauna can be developed if the mesocosm system has an interior metal frame with four legs instead of an exterior wood frame. Thus, the mesh cylinder can be closed at the top making the top cover mesh and the fixing velcros unnecessary.

The second challenge was to provide similar conditions for plants in defaunated and control mesocosms, and a great emphasis should be placed on it. In former microcosm experiments, plant seedlings were growing in controlled conditions (Liiri et al., 2012), which is not valid in the field. In experiment #1, spring wheat could not properly grow in defaunated mesocosms. However, mixed grass developed similarly in the two mesocosms in sandy soil. In experiment #2, the covering mesh net was changed to muslin textile, which was more similar to the 35‐μm mesh net in light filtering but allowed animals to pass through. In addition, control mesocosms needed to be covered with a mesh material similar to defaunated mesocosms because this covering mesh partly hampers the precipitation from falling into the mesocosms and provides similar temperature and humidity conditions inside the semi‐closed system. Without this, the precipitation entering the mesocosms and the air humidity inside were not equal in the two systems. However, in chernozem soil, there was a difference in grass biomass between the treated and control systems. At this location, mixed grass did not properly grow in the control systems. We assume that the reason was the inadequate seeding technique because mixed grass grew similarly in the two systems in the next year (2021, data not shown). In addition, grass biomass can be harvested several times without destroying the plant. Therefore, mixed grass seems to be a better experimental plant in these semi‐closed systems.

The third challenge was to extract soil water samples by using percolation lysimeters. This lysimeter type is a simple and effective tool to collect gravitational soil water under a shallow soil layer in the field (Derome et al., 1991). In laboratory microcosms, percolated water is collected at the bottom of the small‐sized microcosm vessels (Bardgett & Chan, 1999; Bardgett et al., 1998; Cole et al., 2004; Heneghan & Bolger, 1998; Schon et al., 2011). Drainage lysimeters are usually used in the field (Singh et al., 2018). These lysimeters used in agrochemical experiments require large soil monoliths and special underground facilities with high space and cost demands (Bender & van der Heijden, 2015). In contrast, percolation lysimeters need no energy and have low costs. However, if we want to collect adequate quality and quantity of water, lysimeters must be emptied time after time. Fresh and authentic samples can be taken only after substantial rainfall or after direct irrigation. Our experiment shows that these percolation lysimeters work well for at least 8 months (even 18 months, according to recent results) in agricultural fields in sandy and chernozem soil types. However, the percolation lysimeter can be improved in two ways. First, instead of using only nylon mesh inside the funnel, we suggest to use a water‐proof, truncated cone formed nylon having a nylon mesh cone in its top. This composite nylon funnel should be larger than the plastic funnel, and it can cover the edge of the plastic funnel to avoid any soil particles going into the glass jar. Second, in some occasions, the silicon tube crushed under the weight of the soil and the PVC tube. Therefore, we suggest to cover the silicon tube with a garden hose to avoid crushing.

The present mesocosm experiment was the first step in the developmental process. Although it has its disadvantages, it may become an excellent tool to measure nutrient leaching in soils under the manipulation of soil fauna in the field.

### 4.2 Detangling ecosystem service components: Nitrogen cycling and microarthropods

Based on the previous results that the presence of soil fauna stimulates the release of soil nitrogenous ions (Peña‐Peña & Irmler, 2018; Toyota et al., 2013), we expected that inorganic nitrogen compounds would have a higher concentration in the percolated soil water with more abundant soil microarthropods. Several studies using microcosms (Cole et al., 2004; Pieper & Weigmann, 2008; Wickings & Grandy, 2011) or litter‐bags (Lin et al., 2019; Peña‐Peña & Irmler, 2018) reported the same pattern. This pattern was significant in the chernozem soil of experiment #1–2 and in experiment #2 in sandy soil. The opposite significant patterns (lower ion concentrations with higher abundances) were found in sandy soil in experiment #1. Some results found no effects of microarthropods on nitrogen compounds (Ball et al., 2014; Heneghan et al., 1999). Insignificant trends may be more probable in a field experiment because of the high deviations, but variable results are also typical in microcosm experiments (Heneghan & Bolger, 1998).

The different results of the two locations might be related to the soil types. We hypothesized that soil type would modify the effect of defaunation. There is a difference between nutrient leaching and plant nutrient uptake of chernozem and sandy soils (Foereid et al., 2020), and chernozem soil showed rather such patterns that we expected. However, similar patterns were also reported in humic stagnopodzol (Bardgett & Chan, 1999), in humic brown soil (Cole et al., 2004), and in sandy Terric Anthrosol (Pieper & Weigmann, 2008).

Our second hypothesis, which the stimulating role of soil‐dwelling microarthropods in nitrogen release is more emphasized in nitrogen‐limited systems, has not been proven. Fertilized and unfertilized systems had similar patterns concerning leached nutrient ion concentrations and faunal abundance. Fertilization and fauna treatment showed interaction for water soil nitrate concentration in experiment #1, but this experiment was biased because of the difference in plant biomass (wheat yield) and this interaction did not appear again in experiment #2. However, regardless of defaunation, fertilization affected soil nitrate and ammonium concentrations and leached nitrate concentration but not leached ammonium concentration. Ammonium ions may have nitrified into nitrate ions in the soil over time (Sogn et al., 2018). Osler and Sommerkorn (2007) reported that microarthropods could substantially affect nitrogen cycling in nitrogen‐limited habitats. In the present experiment, the modification of soil nitrogen content through fertilization did not seem to affect the relationship between microarthropod abundance and nitrogen leaching. This may have been possible because the unfertilized systems may have not provided nitrogen‐limited conditions for microbiota, in which soil‐dwelling microarthropods would significantly affect nitrogen metabolism of soil bacteria (Osler & Sommerkorn, 2007).

The patterns mentioned above may have been mainly affected by the different plant biomass between the control and defaunated mesocosms. In experiment #1, spring wheat had lower yields in the defaunated mesocosms. In experiment #2, mixed grass had similar biomass in the two systems in sandy soil, while control mesocosms included lower plant biomass in chernozem soil. Plant biomass may affect soil biota interactions differently between the soil types. In sandy soil (experiment #1), the trends were the opposite in leached ions, which we expected. This trend changed after the plant biomass became similar in the two systems in experiment #2, and we obtained, at the trend level, what we expected (higher ion concentration with higher microarthropod abundance). In chernozem soil, plant biomass may not have affected this pattern. Biomass of plant species in chernozem soil was the opposite in the two experiments (#1: higher biomass in control and #2: higher biomass in defaunated mesocosms), and leached ions tended to have or significantly had higher values in the control than in defaunated mesocosms. This result indicates that our second hypothesis was more applicable to chernozem soil almost independently of plant species and biomass. This is one of the main aspects, which will be tested in future in our experiments. However, it is necessary to use plants in such mesocosms. Microcosm studies usually lack growing plants, but the presence of a plant significantly affects the soil nutrient cycling processes (Bardgett et al., 1993).

We expected higher nitrogen uptake by plants with higher soil‐dwelling microarthropod abundance, which was performed in other studies (Graf et al., 2019; Haase et al., 2008; Partsch et al., 2006). This relationship was investigated with spring wheat in experiment #1. In unfertilized chernozem soil, we found what we expected. Meanwhile, wheat plants had higher nitrogen contents in defaunated systems in sandy soil. However, the relation between fauna and plant nitrogen uptake could not be adequately investigated due to the significant yield differences between the control and defaunated mesocosms. This yield difference may not be due to defaunation but to the build‐up differences between control and defaunated mesocosms (see Section 4.1). When the subsequently developed mesocosms provide similar conditions for plants, nitrogen uptake will be comparable among treatments with similar plant biomass.

The soil microbial biomass was estimated by substrate‐induced respiration. We presumed that a higher estimated microbial biomass would be detected with more abundant soil microarthropods. Substrate‐induced respiration values showed this pattern only in unfertilized control mesocosms in chernozem soil. Higher abundances or diversities of soil‐dwelling microarthropods were reported with higher biomass of soil microbiota in several studies (Potapov et al., 2017; Vedder et al., 1996). However, other studies reported that the soil microbiota increases only its enzyme activity (Cole & Bardgett, 2002) or has no change in their communities after microarthropod abundance manipulation (Kaneda & Kaneko, 2011). In the present study, the lowest soil inorganic nitrogen‐ion concentration was measured in the unfertilized control chernozem soil with higher microbiota and microarthropod abundance. Microarthropods may have stimulated microbiota only in these nutrient‐poorer mesocosms (Osler & Sommerkorn, 2007).

## 5 CONCLUSIONS

We developed a field mesocosm system that simultaneously grows plants and keeps a low abundance of soil‐dwelling microarthropods. In addition, the soil solution could be sampled from these systems. Although the mesocosms need further improvement, some of the challenges of field mesocosms have been resolved. This type of field experiment provides considerable opportunities to investigate the role of soil‐dwelling arthropods in ecological processes in the field.

Despite the imperfections of the mesocosms, some of our hypotheses were supported in different experiments, mainly in chernozem soil, not in sandy soil. Therefore, the role of soil‐dwelling microarthropods in nitrogen cycling is dependent on soil type. Significant results and trends supported that inorganic nitrogen‐ion leaching is higher by more abundant soil‐dwelling microarthropods in chernozem soil. However, this pattern was not influenced by a nitrogen addition, so the lack of fertilization presumably did not cause any real nitrogen limitation.

## AUTHOR CONTRIBUTIONS

**Veronika Gergócs:** Conceptualization (lead); data curation (lead); formal analysis (lead); funding acquisition (lead); investigation (lead); methodology (lead); project administration (lead); software (lead); supervision (lead); validation (lead); visualization (lead); writing – original draft (lead); writing – review and editing (lead). **Norbert Flórián:** Conceptualization (supporting); investigation (supporting); methodology (supporting); validation (supporting); writing – review and editing (supporting). **Zsolt Tóth:** Investigation (supporting); methodology (supporting); validation (supporting); writing – review and editing (supporting). **László Sipőcz:** Conceptualization (supporting); investigation (supporting); methodology (supporting); writing – review and editing (supporting). **Miklós Dombos:** Conceptualization (supporting); funding acquisition (supporting); investigation (supporting); methodology (supporting); resources (supporting); supervision (supporting); writing – review and editing (supporting).

## CONFLICT OF INTEREST

None declared.

## Supporting information


Figure S1

Figure S2

Appendix S1

## Data Availability

Soil and water properties and animal abundances will be accessible in Dryad data base: https://doi.org/10.5061/dryad.3ffbg79mp.
