# Supplementary figures and images for: Detangling ecosystem services: Open‐field manipulation of soil‐dwelling microarthropods provides new opportunities to investigate their effects on nitrogen cycling
Source: Ecol Evol. 2022 Jul 19;12(7):e9134. doi: 10.1002/ece3.9134 (PMC9297029; doi:10.1002/ece3.9134)

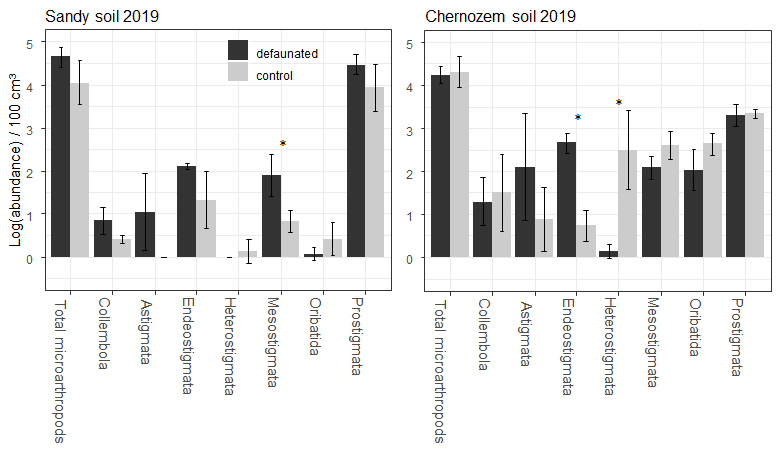

Supplement: Supplementary file 1 — Figure S1 [file ECE3-12-e9134-s001.tif]

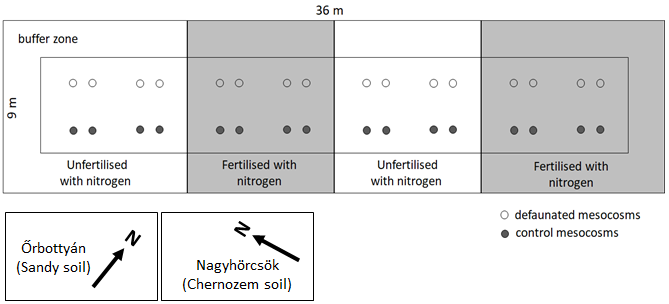

Supplement: Supplementary file 2 — Figure S2 [file ECE3-12-e9134-s002.tif]
