# Supplementary material for: Detangling ecosystem services: Open‐field manipulation of soil‐dwelling microarthropods provides new opportunities to investigate their effects on nitrogen cycling
Source: Ecol Evol. 2022 Jul 19;12(7):e9134. doi: 10.1002/ece3.9134 (PMC9297029; doi:10.1002/ece3.9134)
Supplement: Supplementary file 3 — Appendix S1 [file ECE3-12-e9134-s003.docx]

**Appendix A (Pilot experiment)**

Table S1 Average (± SD) ammonium and nitrate concentrations of the soil water obtained from the pilot experiment.

|  | NH_4_^+^ | NO_3_^-^ |  |
| --- | --- | --- | --- |
| **sandy soil** | mg/l | mg/l | N |
| defaunated | 0.26 ± 0.08 | 136 ± 26 | 4 |
| control | 0.27 ± 0.04 | 139 ± 27 | 4 |
| **chernozem soil** |  |  |  |
| defaunated | 0.51 ± 0.36 | 336.7 ± 31.26 | 4 |
| control | 0.25 ± 0.09 | 310.7 ± 32.58 | 3 |
|  |  |  |  |

Fig. S1 Average (± SD) log-abundances of the microarthropod groups in the two locations of the pilot experiment three months after defaunation. There were significant differences between control and defaunated mesocosms according to the Mann-Whitney-Wilcoxon-test (n=4): *p < 0.05.

**Appendix B (Experiment #1-2)**

Fig.S2 Spatial design for mesocosms in the field (experiments #1-2). Arrows show the north direction.

Table S2 Results of models for microarthropoda abundance data. Mann-Whitney-Wilcoxon model was performed for data in June and July (exp. #1, factor: fertilisation) and two-way ANOVA was performed for data in November (exp. #2, factors: control/defaunated and fertilisation). Abbreviations: W=Mann-Whitney-Wilcoxon test statistic, R^2^ = coefficient of determination, *p < 0.05, **p < 0.01; ***p < 0.001.

|  | June | | July | | November | | | |
| --- | --- | --- | --- | --- | --- | --- | --- | --- |
| **Chernozem soil** | **W** | **p** | **W** | **p** | **R^2^** | **Intercept** | **Fauna** | **Fertilisation** |
| Total microarthropods | 9 | 0.89 | 4 | 0.34 | 0.66 | 1.738*** | 1.880*** | -0.151 |
| Collembola | 8 | 1 | 0 | 0.029* | 0.40 | 0.175 | 0.718*** | 0.056 |
| Astigmata | 11 | 0.47 | 6 | 0.45 | 0.21 | 0.191 | 0.766** | 0.059 |
| Endeostigmata | 3 | 0.19 | 4.5 | 0.38 | 0.23 | 0.548** | 0.636** | 0.265 |
| Heterostigmata | 13 | 0.18 | 5.5 | 0.56 | 0.41 | 0.895** | 1.439*** | -0.177 |
| Mesostigmata | 9 | 0.89 | 6 | 0.69 | 0.27 | 0.042 | 0.520** | 0.086 |
| Oribatida | 11 | 0.46 | 8 | 1 | 0.70 | 0.080 | 2.311*** | 0.105 |
| Prostigmata | 6 | 0.69 | 0 | 0.029* | 0.33 | 0.625*** | 0.789*** | -0.194 |
| **Sandy soil** | **W** | **p** | **W** | **p** | **R^2^** | **Intercept** | **Fauna** | **Fertilisation** |
| Total microarthropods | 6 | 0.69 | 9 | 0.89 | 0.67 | 1.523*** | 1.861*** | 0.177 |
| Collembola | 6 | 0.66 | 13 | 0.19 | 0.68 | 0.341* | 1.402*** | -0.161 |
| Astigmata | 9 | 0.88 | 8.5 | 1 | 0.25 | -0.030 | 0.236** | 0.162 |
| Endeostigmata | 6 | 0.69 | 12 | 0.31 | 0.75 | 0.117 | 1.173*** | -0.026 |
| Heterostigmata | 5 | 0.47 | 9 | 0.88 | 0.52 | 0.857** | 1.646*** | -0.178 |
| Mesostigmata | 8.5 | 1 | 8 | 1 | 0.87 | 0.237* | 1.538*** | -0.192 |
| Oribatida | 6 | 0.65 | 7 | 0.87 | 0.81 | 0.013 | 1.405*** | 0.067 |
| Prostigmata | 3 | 0.19 | 10 | 0.69 | 0.26 | 0.773*** | 0.661** | 0.300 |
